# A Dispensatory, &c.

**Published:** 1849-01

**Authors:** 


### ARTICLE III.

*A Dispensatory, or Commentary on the Pharmacopœias of Great Britain (and the United States); comprising the Natural History, Description, Chemistry, Pharmacy, Actions, Uses, and Doses of the articles of the Materia Medica.* By Robert Christison, M. D., V. P. R. S. E., President of the Royal College of Physicians of Edinburgh, Professor of Materia Medica in the University of Edinburgh, and ordinary Physician to the Queen for Scotland. Second edition, revised and improved, with a supplement, containing the most important new remedies; with copious additions, and two illustrations. By R. Eglesfield Griffith, M. D., author of "A Medical Botany," etc., etc. Philadelphia, Lea & Blanchard, 1848. (From the publishers.)

The above is an excellent work, of 1008 closely printed octavo pages, and will bear a fair comparison with any other dispensatory. This we might expect from the known reputation of the author, who has devoted much of his attention to this branch of medical science. We have carefully examined this work, and can cheerfully testify to its merits. It is, as it purports to be, a "commentary on the phaimacopœias of

Great Britain (and the United States); and an extensive one it is, too; every article being treated of as fully as its importance demands. It gives in detail the natural history, chemistry, and commerce of medicines. All articles of acknowledged utility as medicinal agents are included in the work. Those medicinal plants indigenous to this country, and which did not find a place in the author's edition, have been added to this, by the American editor. The preface to the American edition says: "It will be found to contain very full and copious observations on the various preparations, not only of a theoretical, but also of an eminently practical nature. In the present edition, all the processes of the United States pharmacopœia have been added; and, also, a description of such articles as are recognized in that work, but not noticed by Dr. Christison, with an account of their preparations and uses. Many useful tables and other matter have likewise been added from Redwood's edition of Gray's supplement to the pharmacopœias."

All the late improvements in the pharmacopœias of Great Britain, as well as of the United States, are included; and, also, all that is new in the history, chemistry, and therapeutical application of medicines.

This edition is rendered still more valuable by wood-cuts of medicinal plants, apparatus, &c., with which it is copiously illustrated. It is in all respects brought up to the improvements of the present day.

We can heartily recommend this work as being one of the very best of the kind, and well calculated to supply the wants of the profession.

It makes a good text book for the student, and a reliable work of reference for the pharmaceutist and practicing physician.

